# Initial Experience With Robotic-Assisted Laparoscopic Adrenalectomy in a Tertiary Center: Feasibility, Perioperative Outcomes, and Histopathology

**DOI:** 10.7759/cureus.103564

**Published:** 2026-02-13

**Authors:** Gerardo Rosado Redondo, Miguel-Angel Gonzalez-Rodriguez, Sanjuan Padron-Lucio, Tania-Pilar Alvarez-Dominguez, Ernesto Roldan-Valadez

**Affiliations:** 1 Urology, Centro Médico Naval, Mexico City, MEX; 2 Pathology, Centro Médico Naval, Mexico City, MEX; 3 Research, Instituto Nacional de Rehabilitación “Luis Guillermo Ibarra Ibarra”, Mexico City, MEX; 4 Radiology, I.M. Sechenov First Moscow State Medical University, Sechenov University, Moscow, RUS

**Keywords:** adrenal incidentaloma, clavien-dindo classification, estimated blood loss, intraoperative ultrasonography, length of stay, perioperative outcomes, pheochromocytoma, robotic adrenalectomy, robotic-assisted laparoscopic adrenalectomy, tertiary care center

## Abstract

Introduction: Robotic-assisted laparoscopic adrenalectomy (RALA) extends minimally invasive adrenal surgery by adding three-dimensional visualization and wristed instrumentation. While laparoscopic adrenalectomy remains the standard approach, real-world perioperative performance of RALA in tertiary referral settings warrants evaluation. We aimed to describe the feasibility, perioperative outcomes, complications, and histopathology of consecutive patients undergoing RALA at our institution.

Materials and methods: We conducted a retrospective observational analysis of consecutive patients undergoing RALA at a tertiary referral center (November 2021-November 2024). Demographic, imaging, operative, and pathologic variables were collected. Complications were graded using the Clavien-Dindo system, with patients without events explicitly reported as Grade 0. Analyses were descriptive.

Results: Sixteen patients underwent RALA (10/16 (62.5%) male); median age was 63 years (range 40-83). Median radiologic tumor size was 5.03 cm (range 1.2-12.5). All cases were completed without conversion. Median operative time was 147 minutes (range 70-270), and median estimated blood loss was 105 mL (range 20-250). Postoperative morbidity was low: Grade 0 in 15/16 (93.8%) and one Grade II event in 1/16 (6.2%); no Grade I or Grade III-IV events occurred. Median hospital stay was 7.5 days (range 2-44) overall and three days when excluding a prespecified outlier with bilateral disease and significant comorbidity. Because one patient had bilateral surgery, pathology was reported per lesion (n=17): benign 14/17 (82.4%), malignant 2/17 (11.8%), and indeterminate 1/17 (5.9%); malignancy was suspected preoperatively in one lesion and was an incidental final-pathology finding in the second. Intraoperative ultrasonography was used in one complex case to assist localization.

Discussion: These findings align with contemporary reports of low morbidity and reliable completion for RALA, with operative metrics and recovery profiles within published ranges. Technical elements such as early adrenal vein control may support hemodynamic stability in hormonally active tumors, and selective intraoperative ultrasonography can aid dissection when anatomy is distorted.

Conclusion: In this early institutional series, RALA was feasible and safe, with zero conversions, low blood loss, and very low complication rates, alongside rapid recovery after sensitivity adjustment for a single outlier. Prospective comparative studies are needed to refine patient selection and to evaluate long-term oncologic outcomes and cost-effectiveness.

## Introduction

Laparoscopic adrenalectomy is the standard approach for most functional and nonfunctional adrenal tumors because it reduces blood loss, shortens recovery, and lowers postoperative morbidity compared with open surgery [1]. Robotic-assisted laparoscopic adrenalectomy (RALA) builds on these advantages by adding three-dimensional visualization, tremor filtration, and wristed instrumentation that can facilitate precise dissection and vascular control in confined operative fields [2]. Since the first reports in the late 1990s, multiple centers have demonstrated the feasibility and safety of robotic adrenalectomy across a broad spectrum of adrenal pathology [3-5].

In parallel, the widespread use of contrast-enhanced computed tomography (CT) has increased detection of adrenal incidentalomas, prompting structured endocrine and radiologic workups to define hormonal function and malignancy risk [6,7]. In technically demanding scenarios, larger lesions, richly vascular tumors such as pheochromocytoma, or dense adhesions, robotic technology may offer practical advantages by improving dexterity and exposure during fine dissection [8-10]. However, debate persists regarding cost-effectiveness and the magnitude of benefit over conventional laparoscopy, particularly for operative efficiency and oncologic endpoints [6].

Within this context, we aimed to describe our institution’s initial experience with RALA in a tertiary care setting. Specifically, we report feasibility outcomes (completion without conversion), perioperative performance (operative time, estimated blood loss, time to oral intake, length of stay (LOS)), postoperative morbidity graded by Clavien-Dindo, and final histopathology in consecutive cases, providing real-world benchmarks to inform clinical decision-making and guide future comparative studies [11-13].

## Materials and methods

Study design and setting

We conducted a retrospective observational study at Centro Médico Naval, a tertiary referral hospital in Mexico City. Consecutive patients undergoing RALA between November 2021 and November 2024 were analyzed in accordance with STROBE (STrengthening the Reporting of OBservational studies in Epidemiology) principles. Data were abstracted from electronic medical records and anonymized per institutional standards.

Patient selection and eligibility 

The cohort comprised 16 consecutive patients who underwent RALA for adrenal tumors during the study period. Preoperative workup included contrast-enhanced CT and multidisciplinary assessment by endocrinology and urology to characterize hormonal function and determine surgical eligibility. To limit selection bias, all consecutive eligible patients within the time window were included; two investigators independently extracted data and resolved discrepancies by consensus. Because this was a descriptive series, no a priori sample-size calculation was planned.

Inclusion criteria

Radiologically confirmed adrenal tumor selected for surgery by the multidisciplinary team; maximal diameter ≤12 cm on preoperative CT; American Society of Anesthesiologists (ASA) class II-III.

Exclusion criteria

Prior adrenal surgery; radiologic evidence of distant metastases or local invasion precluding a minimally invasive approach; severe cardiopulmonary disease contraindicating pneumoperitoneum.

Preoperative evaluation and imaging

All patients underwent contrast-enhanced CT as the primary imaging modality. When available, attenuation values were recorded on unenhanced images, and enhancement behavior was documented on contrast phases; delayed images were used when washout was assessed. Endocrinologic evaluation was performed when clinical or biochemical suspicion suggested functional disease. Imaging characteristics (lesion size, laterality, and salient features) were recorded for operative planning.

Perioperative management

Patients received general anesthesia with thromboembolism and antibiotic prophylaxis per institutional protocol. For suspected catecholamine-secreting tumors, preoperative adrenergic blockade and hemodynamic optimization were undertaken in coordination with anesthesia and endocrinology, and early adrenal venous control was planned. Postoperative discharge readiness generally required clinical stability, adequate pain control with oral analgesics, tolerance of oral intake, and ambulation, consistent with routine institutional practice.

Surgical technique

All operations were performed by the same senior endocrine surgeon using the da Vinci Xi platform. Patients were positioned in lateral decubitus with protective padding. A five-port transperitoneal approach was used, including one assistant port. Key steps included entry to the retroperitoneum; identification and early ligation of adrenal vessels; mobilization and en bloc gland resection; specimen retrieval in an endoscopic bag; and meticulous hemostasis with layered port-site closure. In one complex case of suspected adrenal metastasis from hepatocellular carcinoma, intraoperative ultrasonography (BK5000) aided localization and guided resection.

Outcomes and definitions

Collected variables included demographics (age, sex, body mass index [BMI], ASA class); surgical parameters (laterality, radiologic tumor size, operative time, estimated blood loss, conversion); postoperative recovery (time to oral intake, length of stay); complications; and histopathology. Operative time was defined from skin incision to skin closure; conversion was defined as any unplanned open procedure. Length of stay (LOS) was defined as days from the calendar date of surgery to hospital discharge. Complications within 30 days were graded using the Clavien-Dindo classification [14], a standardized framework that categorizes postoperative morbidity according to the therapy required; to enhance transparency and facilitate benchmarking across published series, patients without postoperative events were explicitly reported as Grade 0, and Grades I-IV denoted increasing severity [15]. Because one patient underwent bilateral adrenalectomy, histopathology was summarized per lesion (n=17) and categorized as benign, malignant, or indeterminate.

Statistical analysis

Analyses were descriptive and performed in SPSS v25 (IBM Inc., Armonk, New York). Categorical variables are summarized as n (%), with numerator and denominator specified when helpful. Continuous variables are summarized as medians with ranges, or medians with interquartile ranges where indicated. Table and figure legends state the summary measure used for each variable. Percentages for patient-level variables use the cohort denominator of n=16; histopathology is summarized per lesion because one patient had bilateral disease (n=17). No formal hypothesis testing was planned or performed because this was a single-arm, small-sample retrospective series; accordingly, p-values and test statistics are not presented. If inferential comparisons are undertaken in future analyses, statistical significance would be defined a priori as two-sided α=0.05. As prespecified, LOS is presented overall and in a sensitivity analysis excluding the single bilateral case with prolonged hospitalization due to preexisting hepatic disease.

Ethical considerations

The Institutional Review Board of Centro Médico Naval approved the study and waived informed consent owing to its retrospective design (approval No. CMN022/2025). Procedures conformed to the Declaration of Helsinki; all data were anonymized prior to analysis.

## Results

Patient demographics and clinical characteristics

Sixteen consecutive patients underwent RALA between November 2021 and November 2024. Median age was 63 years (range 40-83) and median BMI was 25.4 kg/m² (range 17.6-32.9). The cohort included 10/16 (62.5%) male and 6/16 (37.5%) female patients, and the ASA class was predominantly II-III. Baseline characteristics are summarized in Table 1.

**Table 1:** Baseline demographic and clinical characteristics of patients undergoing RALA. Continuous variables are reported as median (range); categorical variables are reported as n (%). The patient-level denominator is n=16. No hypothesis testing was performed; p-values are not applicable. RALA, robotic-assisted laparoscopic adrenalectomy; ASA, American Society of Anesthesiologists

Variable	Value
Age at surgery, years	63 (40-83)
Male sex, n (%)	10 (62.5)
Female sex, n (%)	6 (37.5)
Body mass index, kg/m²	25.4 (17.6-32.9)
ASA class, median (range)	3 (2-3)

Tumor characteristics and operative metrics

Laterality was left in 8/16 (50.0%), right in 7/16 (43.8%), and bilateral in 1/16 (6.2%). Median radiologic tumor size was 5.03 cm (range 1.2-12.5). All procedures were completed without conversion to open surgery. Median operative time was 147 minutes (range 70-270), and median estimated blood loss was 105 mL (range 20-250). Median time to oral intake was 18 hours (range 6-30). Median LOS was 7.5 days (range 2-44); excluding a prespecified outlier (bilateral case with preexisting hepatic disease), median LOS was three days (Table 2).

**Table 2:** Surgical and perioperative outcomes of patients undergoing RALA. Continuous variables are reported as median (range); categorical variables are reported as n (%). The patient-level denominator is n=16. Patients without postoperative events were explicitly reported as Clavien-Dindo Grade 0. No hypothesis testing was performed; p-values are not applicable. Note: LOS includes a prolonged hospitalization for the single bilateral case with preexisting hepatic disease; excluding this prespecified outlier, median LOS was three days. RALA, robotic-assisted laparoscopic adrenalectomy; LOS, length of stay

Variable	Value
Radiologic tumor size, cm	5.03 (1.2-12.5)
Right-sided lesion, n (%)	7 (43.8)
Left-sided lesion, n (%)	8 (50.0)
Bilateral lesions, n (%)	1 (6.2)
Operative time, minutes	147 (70-270)
Estimated blood loss, mL	105 (20-250)
Time to oral intake, hours	18 (6-30)
Length of hospital stay, days	7.5 (2-44)
Intraoperative complications, n (%)	0 (0.0)
Postoperative events within 30 days, n (%)	1 (6.2)
Clavien–Dindo Grade 0, n (%)	15 (93.8)
Clavien–Dindo Grade I, n (%)	0 (0.0)
Clavien–Dindo Grade II, n (%)	1 (6.2)
Clavien–Dindo Grade III, n (%)	0 (0.0)
Clavien–Dindo Grade IV, n (%)	0 (0.0)

Perioperative complications and recovery

There were no intraoperative complications. Within 30 days, postoperative events occurred in 1/16 patients (6.2%). Using the Clavien-Dindo system with explicit tabulation of patients without events as Grade 0, the distribution was Grade 0 in 15/16 (93.8%), Grade I in 0/16 (0%), Grade II in 1/16 (6.2%), and Grades III-IV in 0/16 (0%) (Table 2). Most patients resumed oral intake within 24 hours, consistent with the short LOS observed after sensitivity adjustment.

Histopathological findings

Because one patient had bilateral disease, there were 17 adrenal lesions in 16 patients. Pathology by lesion was benign in 14/17 (82.4%), malignant in 2/17 (11.8%), and indeterminate in 1/17 (5.9%). Malignancy was suspected preoperatively in 1/2 lesions based on imaging and clinical context, whereas the second malignant diagnosis was established incidentally on final histopathology. The most frequent benign subtypes were adrenal cortical adenoma in 5/17 (29.4%), pheochromocytoma in 3/17 (17.6%), and myelolipoma in 3/17 (17.6%); other entities included endothelial/lymphangiomatous cysts, nodular cortical hyperplasia, and one oncocytic neoplasm of uncertain malignant potential (Table 3).

**Table 3:** Histopathological classification and tumor subtypes of adrenal lesions resected via RALA. Continuous variables are reported as median (range); categorical variables are reported as n (%). Percentages are per lesion because one patient had bilateral disease (n=17). No hypothesis testing was performed; p-values are not applicable. RALA, robotic-assisted laparoscopic adrenalectomy

Variable	Value
Pathologic specimen size, cm	5.51 (1.2-12.5)
Pathology classification, n (%)	
Malignant	2 (11.8%)
Benign	14 (82.4%)
Indeterminate	1 (5.9%)
Final histological characteristics, n (%)	
Adrenal cortical adenoma	5 (29.4%)
Multiloculated calcified endothelial lymphangiomatous cyst	1 (5.9%)
Secondary tumor metastasis	2 (11.8%)
Pheochromocytoma	3 (17.6%)
Oncocytic neoplasm of uncertain malignant potential	1 (5.9%)
Myelolipoma	3 (17.6%)
Nodular cortical hyperplasia	1 (5.9%)
Endothelial angiomatous cyst	1 (5.9%)

Imaging-guided surgical enhancement and representative cases

**Figure 1:**
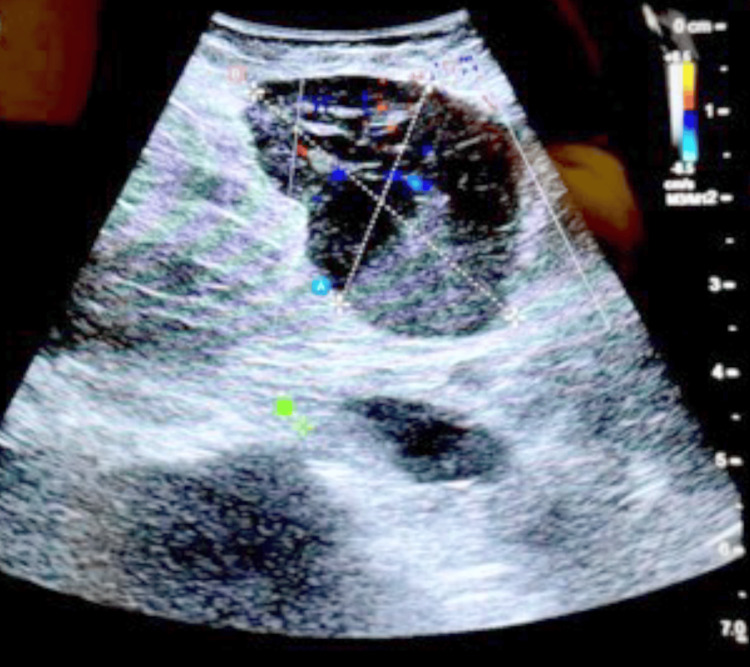
Intraoperative real-time ultrasonography (BK5000; BK Medical) during RALA in a patient with suspected adrenal metastasis from hepatocellular carcinoma. Ultrasonography delineated lesion margins and adjacent vascular structures, improving localization and guiding precise dissection. Representative single-patient image (no statistical comparison performed). RALA, robotic-assisted laparoscopic adrenalectomy

**Figure 2:**
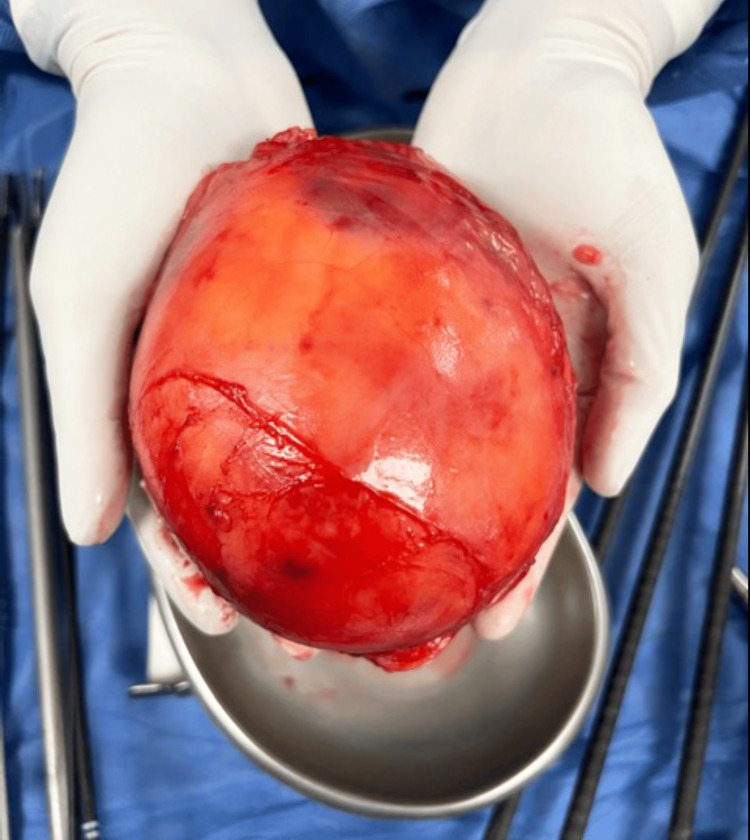
Gross specimen of a large adrenal mass (12.5×11×7 cm) retrieved after RALA. Final histopathology confirmed myelolipoma. Representative single-patient image (no statistical comparison performed). RALA, robotic-assisted laparoscopic adrenalectomy

**Figure 3:**
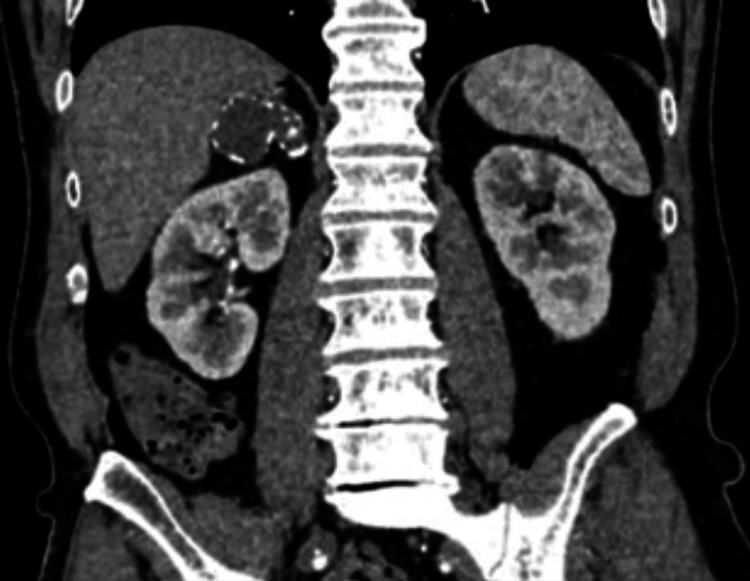
Coronal contrast-enhanced abdominal CT demonstrating a 51×40 mm adrenal lesion with attenuation of 45 HU. The attenuation and enhancement pattern supported a benign etiology and informed preoperative planning. Representative single-patient image; values shown are direct measurements from the depicted case (no statistical comparison performed). CT, computed tomography

**Figure 4:**
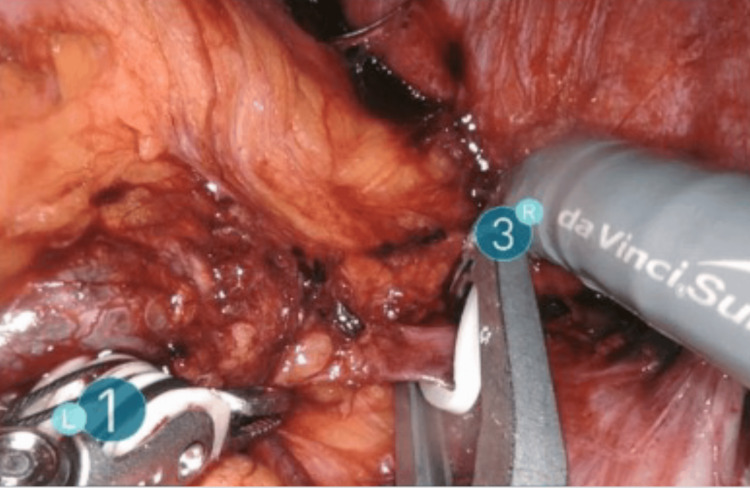
Intraoperative view of early adrenal vein ligation during RALA for pheochromocytoma. Early venous control is used to mitigate catecholamine surges and reduce intraoperative bleeding risk. Representative technique image (no statistical comparison performed). RALA, robotic-assisted laparoscopic adrenalectomy

In a patient with suspected adrenal metastasis from hepatocellular carcinoma, intraoperative ultrasonography (BK5000) facilitated lesion localization during RALA (Figure 1). A representative large myelolipoma (12.5×11×7 cm) excised robotically is shown in Figure 2. Figure 3 depicts a contrast-enhanced CT example used for preoperative planning, and Figure 4 illustrates early adrenal vein control during pheochromocytoma resection.

## Discussion

Principal findings

This single-center experience supports the feasibility and short-term safety of robotic-assisted laparoscopic adrenalectomy (RALA) in a tertiary setting. Among 16 consecutive patients, there were no conversions to open surgery, the median estimated blood loss was 105 mL, and postoperative morbidity was low: 15/16 patients had no events (Clavien-Dindo Grade 0), and one experienced a Grade II event. These benchmarks align with contemporary series reporting low morbidity and reliable completion rates for RALA in appropriately selected patients [1-4,11,12,16,17].

Comparison with recent literature and operative performance

Operative efficiency in this early program fell within published ranges. Median operative time was 147 minutes overall; unilateral procedures clustered near 140 minutes when the bilateral case was excluded, consistent with transperitoneal RALA benchmarks reported by high-volume and multicenter experiences [11-13,16]. The technical features of robotic platforms-stable three-dimensional visualization, tremor filtration, and wristed instrumentation-may facilitate meticulous dissection and vascular control in confined operative fields, which has been emphasized in prior reports and reviews [2-4,6].

Recovery and length of stay

Patients generally resumed oral intake within 24 hours. Median LOS was 7.5 days overall; excluding a prespecified outlier (bilateral adrenalectomy with significant pre-existing hepatic disease), LOS fell to 3 days. Reporting both values improves interpretability in early-adoption series where a single complex course can disproportionately influence summary estimates. These recovery patterns are consistent with minimally invasive adrenalectomy literature describing early return of bowel function and short hospitalization in uncomplicated cases [3,12,17,18].

Technical notes: early adrenal vein control and intraoperative ultrasonography Early ligation of the adrenal vein remains a cornerstone of safe adrenalectomy, particularly for pheochromocytoma, where catecholamine surges can destabilize hemodynamics [2,4,6,9]. Our pathway emphasized coordinated anesthetic management and early venous control; no intraoperative hypertensive crises occurred in functional tumors in this series. Pharmacologic adjuncts such as clevidipine have been explored to enhance hemodynamic stability during adrenalectomy and may complement this strategy in selected cases, although comparative effectiveness requires further study [19].

In a complex case with suspected metastatic disease, real-time intraoperative ultrasonography (BK5000) improved lesion localization and supported dissection when standard visual cues were limited. As robotic programs mature, selective integration of intraoperative imaging may be useful when planes are distorted, margins are uncertain, or vascular anatomy requires additional confirmation.

Histopathology and case mix

Because one patient had bilateral disease, histopathology was reported per lesion (n=17): benign 14/17 (82.4%), malignant 2/17 (11.8%), and indeterminate 1/17 (5.9%). This distribution reflects contemporary practice in the era of incidental detection, with cortical adenoma, pheochromocytoma, and myelolipoma predominating, while the malignant lesions underscore the value of rigorous preoperative imaging, endocrine assessment, and multidisciplinary selection. For benign cystic lesions, contemporary data provide additional context regarding imaging features and clinical course [16].

Clinical implications and future research

These early results suggest that RALA can be implemented as a dependable minimally invasive option in tertiary care, including technically demanding scenarios such as larger lesions or hormonally active tumors, provided appropriate expertise and perioperative coordination are in place. Future work should prioritize prospective comparisons with standard laparoscopy, learning-curve evaluation, standardized hemodynamic endpoints in pheochromocytoma, cost-effectiveness analyses, patient-reported outcomes, and longer-term oncologic endpoints in lesions with malignant potential [12,13,17-19].

Limitations

The retrospective, single-center design and small sample size limit precision and external validity. There was no contemporaneous laparoscopic comparator, so claims of superiority are unwarranted. In addition, standardized cost data and long-term oncologic outcomes were not assessed, and endocrine outcomes for functional tumors were not systematically captured.

Overall interpretation

Taken together, these findings provide pragmatic early-program benchmarks demonstrating that RALA was completed without conversion and with low short-term morbidity in this consecutive series. The results support continued program development and motivate larger, comparative studies to refine patient selection, quantify resource utilization, and better define long-term outcomes.

## Conclusions

In this early single-center series, RALA was completed without conversion and with low short-term morbidity: 15/16 patients (93.8%) had no postoperative events (Clavien-Dindo Grade 0), and 1/16 (6.2%) experienced a Grade II event. Median LOS was 7.5 days overall and three days after excluding a prespecified outlier (bilateral adrenalectomy with significant comorbidity), and most lesions were benign on final histopathology.

Intraoperative ultrasonography served as a useful adjunct in a complex case requiring improved lesion localization. Taken together, these real-world perioperative outcomes support RALA as a feasible option in tertiary care and justify larger comparative studies to refine patient selection, quantify resource utilization and cost-effectiveness, and define longer-term oncologic outcomes.
